# Effects of carbohydrate, branched-chain amino acids, and arginine in recovery period on the subsequent performance in wrestlers

**DOI:** 10.1186/1550-2783-8-21

**Published:** 2011-11-22

**Authors:** Tsong-Rong Jang, Ching-Lin Wu, Chai-Ming Chang, Wei Hung, Shih-Hua Fang, Chen-Kang Chang

**Affiliations:** 1 Department of Combat Sports, National Taiwan College of Physical Education, 16, Sec 1, Shuan-Shih Rd, Taichung, 404, Taiwan; 2 Graduate Institute of Sports and Health Management, National Chung Hsing University, 250 Kuo Kuang Road, Taichung, 402, Taiwan; 3 Institute of Athletics, National Taiwan College of Physical Education, 16, Sec 1, Shuan-Shih Rd, Taichung, 404, Taiwan; 4 Department of Exercise Health Science, National Taiwan College of Physical Education, 16, Sec 1, Shuan-Shih Rd, Taichung, 404, Taiwan; 5 Sport Science Research Center, National Taiwan College of Physical Education, 16, Sec 1, Shuan-Shih Rd, Taichung, 404, Taiwan

**Keywords:** high-intensity intermittent exercise, insulinemic, exercise performance, exercise recovery

## Abstract

Many athletes need to participate in multiple events in a single day. The efficient post-exercise glycogen recovery may be critical for the performance in subsequent exercise. This study examined whether post-exercise carbohydrate supplementation could restore the performance in the subsequent simulated wrestling match. The effect of branched-chain amino acids and arginine on glucose disposal and performance was also investigated. Nine well-trained male wrestlers participated in 3 trials in a random order. Each trial contained 3 matches with a 1-hr rest between match 1 and 2, and a 2-hr rest between match 2 and 3. Each match contained 3 exercise periods interspersed with 1-min rests. The subjects alternated 10-s all-out sprints and 20-s rests in each exercise period. At the end of match 2, 3 different supplementations were consumed: 1.2 g/kg glucose (CHO trial), 1 g/kg glucose + 0.1 g/kg Arg + 0.1 g/kg BCAA (CHO+AA trial), or water (placebo trial). The peak and average power in the 3 matches was similar in the 3 trials. After the supplementation, CHO and CHO+AA trial showed significantly higher glucose and insulin, and lower glycerol and non-esterified fatty acid concentrations than the placebo trial. There was no significant difference in these biochemical parameters between the CHO and CHO+AA trials. Supplementation of carbohydrate with or without BCAA and arginine during the post-match period had no effect on the performance in the following simulated match in wrestlers. In addition, BCAA and arginine did not provide additional insulinemic effect.

## Introduction

Carbohydrate availability is one of the crucial factors for performance in endurance [1] and high-intensity intermittent exercise [2]. It has been well-documented that carbohydrate supplementation before a single-bout of endurance [3] and high-intensity intermittent exercise [4] could improve the performance. In real circumstances, many athletes undergo more than 1 training session per day. In addition, many competitions require athletes to participate in multiple events in a single day. Therefore, adequate nutritional strategies during the short-term post-exercise recovery period may be critical for the performance in subsequent exercise. Several studies have shown that ingestion of protein with carbohydrate after exercise increases muscle glycogen resynthesis rate, compared to the same amount of carbohydrate [5,6]. The increased muscle glycogen recovery may lead to the improved performance during subsequent endurance exercise [7].

Muscle glycogen resynthesis after exercise consists of two phases. The initial insulin-independent phase that lasts approximately 1 hour has a higher resynthesis rate. It is followed by an insulin-dependent phase with a lower rate that lasts several hours [8]. Previous studies have suggested that branched-chain amino acids (BCAA) and arginine may help improve both phases. Studies in rats have shown that BCAA could stimulate insulin-independent glucose uptake in skeletal muscle by increasing the translocation of glucose transporter (GLUT)-4 and GLUT-1 to the sarcolemma [9]. Leucine also activated glycogen synthetase via activation of mammalian target of rapamycin (mTOR) signals in isolated muscles [10]. Isoleucine increased insulin-independent glucose uptake and glycogen synthesis in C_2_C_12 _myotubes [11]. In addition, nitric oxide (NO), a product of arginine, could increase the insulin-independent expression and translocation of GLUT-4 in rat skeletal muscles [12]. The vasodilation effect of arginine could increase blood flow and substrate delivery to the muscle and further increase glycogen recovery [13].

BCAA and arginine may also facilitate the insulin-dependent phase by inducing insulin secretion [14,15]. The consumption of leucine and arginine along with glucose could result in higher insulinemic response compared to glucose alone in healthy subjects at rest [16]. In addition, the supplementation of leucine in combination with carbohydrate resulted in higher post-exercise insulin concentration and greater muscle glycogen recovery compared to the same amount of carbohydrate in athletes [5,17]. Arginine supplementation after endurance exercise could also increase glucose and insulin concentrations during the recovery period in trained athletes [18]. Another study revealed that arginine increased insulin-mediated whole-body glucose disposal in healthy subjects [19], which might help to increase post-exercise glycogen resynthesis. On the other hand, a study using isotope-labeled glucose revealed that protein hydrolysate with or without leucine had no effect on post-exercise glucose disposal, compared to the same amount of carbohydrate, despite higher insulinemic responses [20].

Wrestling is a sport characterized by high-intensity bouts interspersed with brief periods of mild- to moderate-intensity work or rest [21]. Olympic and international wrestling events require athletes to compete in multiple matches in one day. The rest between matches are usually 1-3 hrs. It has been shown that a free-style wrestling match decreased the glycogen level in the vastus lateralis muscle by 21.5% [22]. Several studies have reported post-match blood lactate concentration at 10.5-20 mM [22-25], indicating that carbohydrate is the major energy source in wrestling. If appropriate nutrition/supplementation is not taken, it is hypothesized that the low muscle glycogen level resulted from previous matches would impair the performance in the subsequent match. Therefore, this study investigated the effects of 2 isocaloric supplements, carbohydrate or carbohydrate plus BCAA and arginine, consumed during the post-match recovery period on the performance in the subsequent match in well-trained college wrestlers. The purpose was two-fold: to examine (1) whether carbohydrate supplementation could restore the performance and (2) whether BCAA and arginine could provide additive effect on glucose disposal during the recovery and the performance in the subsequent match.

## Material and methods

### Subjects

Nine well-trained male wrestlers were recruited from National Taiwan College of Physical Education, Taichung, Taiwan. Their age was 19.2 ± 0.4 (mean ± SEM) years, the height was 1.69 ± 0.02 m, the body weight was 72.18 ± 2.71 kg, the body fat was 15.5 ± 1.6%, and $\dot{\text{V}}$ O_2max _was 55.5 ± 1.0 ml/kg/min. The subjects were free of known cardiovascular disease risks and musculoskeletal injuries. The subjects had not taken any protein supplement in the previous 3 months. All subjects have undergone regular wrestling training for at least 4 years and competed in national or international level. The subjects were asked to maintain their regular training schedule and diet habits during the study period, except on the day before each trial when all training was avoided. All subjects gave their written informed consent after the experimental procedure and potential risks were explained. The study protocol was approved by the Human Subject Committee of National Taiwan College of Physical Education.

### Study design

**Figure 1:**
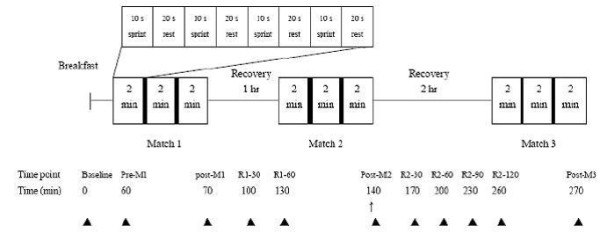
**Study protocol**. ■: 1-min rest; ↑: supplementation; ▲: blood sampling.

This study used a double-blind, randomized cross-over design. The procedure of exercise tests and blood and gas samplings is shown in Figure 1. Each subject completed 3 trials in a random order according to their order of admission to this study. Each trial was separated by at least 2 weeks. The same food was provided in the lunch and dinner on the day before, and the breakfast on the day of each trial. The lunch and dinner were meal boxes purchased from a local restaurant. The 2 meals combined to provide approximately 1434 kcal, with 49.7% energy from carbohydrate, 30.1% from fat, and 20.2% from protein. The diet analysis was performed by a dietitian using Taiwanese food exchange table [26]. The breakfast contained white bread 1.2 g/kg, jam 0.1 g/kg, butter 0.l g/kg, and soybean milk 5 ml/kg (6.2 kcal/kg, containing carbohydrate 1.0 g/kg, protein 0.24 g/kg, and fat 0.14 g/kg). For a 70-kg subject, the breakfast contained 434 kcal, including 70 g carbohydrate, 16.8 g protein, and 9.8 g fat.

### Measurement of cardiopulmonary function

The cardiopulmonary function was measured approximately 1-2 weeks prior to the trials using an electrically braked cycle ergometer (ERG 550, Bosch, Stuttgart, Germany). The subjects warmed up at 50 W for 5 min, followed by incremental steps of 25 W every 3 min. The breath-by-breath gas analysis was performed using a gas analyzer (Vmax 29C, Sensormedics, Yorba Linda, CA, USA). The $\dot{\text{V}}$ O_2max _was considered to be achieved if $\dot{\text{V}}$ O_2 _increased by no more than 2 ml/kg/min after increasing the workload or a respiratory exchange ratio was larger than 1.10.

### Experimental procedure

The subjects reported to the laboratory in the early morning after an overnight fast. A cannula was put in the antecubital vein by licensed personnel. After a blood sample was taken in a fasted state to serve as the baseline, the subjects consumed the standardized breakfast. The exercise test started 1 hr after the breakfast was consumed. Each trial contained 3 matches. At the end of the second match, 3 different supplementations were consumed: 1.2 g/kg glucose (CHO trial), 1 g/kg glucose + 0.1 g/kg Arg + 0.1 g/kg BCAA (leucine: isoleucine: valine = 2:1:1, CHO+AA trial), or water (placebo trial). All supplementations were dissolved in 600 ml lemon flavored water to make the tastes similar. The subjects were allowed to drink water ad libtum in the first trial, while the timing and amount of consumption were recorded. The timing and amount of water consumption were repeated in the following trials.

### Exercise tests

The high-intensity intermittent exercise test was designed to mimic the duration of the actual wrestling competition. The tests were performed on a Monark cycle ergometer (894E, Monark, Varberg, Sweden). Each trial contained 3 matches with a 1-hr rest between match 1 and 2 and a 2-hr rest between match 2 and 3. A match contained 3 exercise periods lasting 2 minutes each with a work to rest ratio of 10 seconds: 20 seconds. After each exercise period, a 2 minute rest period was provided before the next exercise period. The load was 0.1 kp/kg body weight. The subjects were asked to pedal as fast as possible with vocal encouragement by research personnel. In the rest periods the load was removed and the subjects were asked to pedal at 60 rpm. The peak and average power of each sprint was recorded.

### Blood sample collection

Blood samples were collected via an indwelled cannula (20G). The cannula was frequent flushed by sterilized saline to keep it patent throughout the experiment. Ten milliliters of blood sample were collected into an EDTA tube at each sampling time. Hematological analysis was performed immediately after the samples were taken. Thereafter, the rest samples were centrifuged at 1500 × g (Eppendorf 5810, Hamburg, Germany) to extract plasma. The aliquoted plasma samples were stored at -70°C before analysis.

### Biochemical and hormone measurements

The research personnel who conducted the analysis were blind to the group of the samples. Hemoglobin concentration and hematocrit in whole blood was measured by a hematology analyzer (KX-21N, Sysmex Corporation, Kobe, Japan) to correct for the change in plasma volume [27]. Plasma NO_x _concentration was measured with modified Griess reaction using a commercial kit (Sigma, St. Louis, MO, USA). The absorbance at 540 nm was measured with a microplate spectrophotometer (Benchmark Plus, Bio-Rad, Hercules, CA, USA). Plasma concentrations of insulin were measured by electrochemiluminescence (Elecsys 2010, Roche Diagnostics, Basel, Switzerland) with the kit provided by the manufacturer. Plasma glucose, glycerol and non-esterfied fatty acid (NEFA) were measured with an automatic analyzer (Hitachi 7020, Tokyo, Japan) using commercial kits (Randox, Antrim, UK).

### Statistical analysis

All values were expressed as means ± SEMs. The area under the curve (AUC) was calculated for plasma concentrations of glucose and insulin, as well as total carbohydrate and fat oxidation, during the 2-hr recovery period after the second match. The changes in exercise performance, plasma concentrations of metabolites, and substrate oxidation rates were analyzed by a two-way analysis of variance with repeated measures. If the treatment or interaction effect was significant, the differences among the 3 trials at the same time point were identified by post hoc Bonferroni test. The AUC and total carbohydrate and fat oxidation were analyzed by a one-way analysis of variance with repeated measures. If the main effect was significant, the differences among the 3 trials were identified by post hoc Bonferroni test. The analysis was performed with SPSS for Windows 15.0 (SPSS, Chicago, IL, USA). A P value less than .05 was considered statistically significant.

## Results

**Figure 2:**
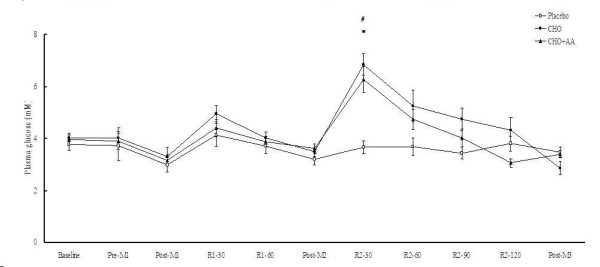
**Plasma glucose concentrations in the 3 trials**. Data were analyzed by using repeated measures ANOVA with time and group as factors. Treatment effect p = 0.006; time effect p < 0.001; interaction effect p < 0.001. *CHO trial significantly different from placebo trial at the same time point (p < 0.05). ^#^CHO+AA trial significantly different from placebo trial at the same time point (p < 0.05).

**Figure 3:**
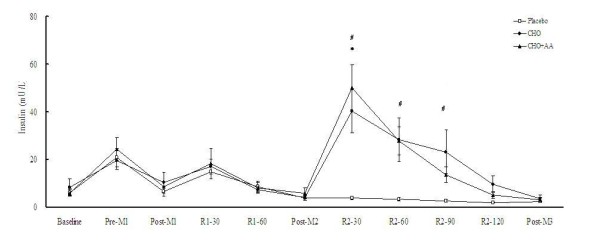
**Plasma insulin concentrations in the 3 trials**. Data were analyzed by using repeated measures ANOVA with time and group as factors. Treatment effect p = 0.013; time effect p < 0.001; interaction effect p < 0.001. *CHO trial significantly different from placebo trial at the same time point (p < 0.05). ^#^CHO+AA trial significantly different from placebo trial at the same time point (p < 0.05).

The peak and average power in the 3 matches was similar in the 3 trials (Table 1). The power drop between match 1 and match 2, as well as between match 1 and match 3, were also similar in the 3 trials. Plasma glucose and insulin concentrations in the 3 trials were shown in Figures 2 and 3, respectively. After supplementations at the end of match 2, the CHO and CHO+AA trial showed significantly higher glucose concentration at 30 min, and significantly higher insulin concentration after 30, 60, and 90 min. Compared to the placebo trial, the CHO and CHO+AA trial also showed significantly higher AUC in glucose (Placebo: 428.69 ± 24.80; CHO: 621.85 ± 41.28; CHO+AA: 550.66 ± 32.89 arbitrary unit; p < 0.01) and insulin concentrations (Placebo: 368.99 ± 68.24; CHO: 2947.01 ± 665.08; CHO+AA: 2896.27 ± 557.40 arbitrary unit; p < 0.01) during the 2-hr recovery period after match 2. However, there was no significant difference between the CHO and CHO+AA trial in either glucose or insulin concentration at any time point. The AUC of plasma glucose and insulin concentrations were also similar between the CHO and CHO+AA trials.

**Table 1:** Peak and average power in 3 matches in the 3 trials^1^

	Placebo trial	CHO trial	CHO+AA trial
Peak power			
1st match (W/kg)	70.36 ± 3.38	71.24 ± 4.19	72.62 ± 4.59
2nd match (W/kg)	69.45 ± 5.40	69.05 ± 5.42	72.08 ± 6.14
3rd match (W/kg)	67.49 ± 4.81	68.72 ± 4.84	72.52 ± 8.18
Average power			
1st match (W/kg)	61.97 ± 3.33	63.90 ± 3.82	64.24 ± 4.14
2nd match (W/kg)	61.41 ± 4.84	61.05 ± 4.59	63.48 ± 5.54
3rd match (W/kg)	59.27 ± 4.15	60.89 ± 4.42	63.85 ± 7.09
Drop in peak power			
Match 1 - Match 2 (%)	1.93 ± 5.07	3.35 ± 4.36	1.49 ± 4.14
Match 1 - Match 3 (%)	4.62 ± 3.93	3.52 ± 3.75	2.17 ± 6.61
Drop in average power			
Match 1 - Match 2 (%)	1.28 ± 5.18	4.58 ± 4.23	2.00 ± 4.14
Match 1 - Match 3 (%)	4.54 ± 4.10	4.65 ± 4.04	2.59 ± 6.45

^1 ^Each trial contained 3 matches with a 1-hr rest between match 1 and 2 and a 2-hr rest between match 2 and 3. A match contained 3 exercise periods lasting 2 minutes each with a work to rest ratio of 10 seconds: 20 seconds. After each exercise period, a 2 minute rest period was provided before the next exercise period. The load was 0.1 kp/kg body weight. All values are means ± SEMs. Data were analyzed by using repeated measures ANOVA with time and group as factors. No significant main effect was observed for any of the variables.

**Figure 4:**
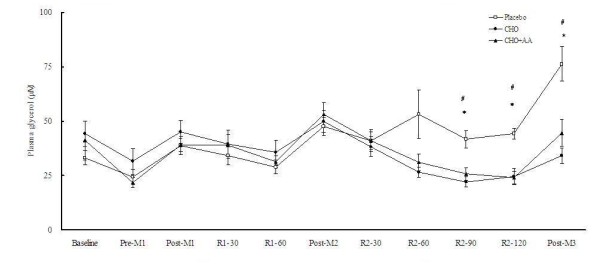
**Plasma glycerol concentrations in the 3 trials**. Data were analyzed by using repeated measures ANOVA with time and group as factors. Treatment effect p = 0.262; time effect p < 0.001; interaction effect p < 0.001. *CHO trial significantly different from placebo trial at the same time point (p < 0.05). ^#^CHO+AA trial significantly different from placebo trial at the same time point (p < 0.05).

**Figure 5:**
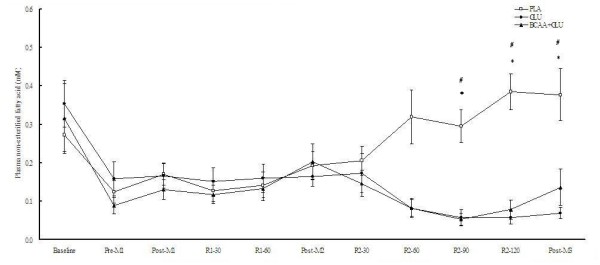
**Plasma non-esterified fatty acid concentrations in the 3 trials**. Data were analyzed by using repeated measures ANOVA with time and group as factors. Treatment effect p = 0.017; time effect p < 0.001; interaction effect p < 0.001. *CHO trial significantly different from placebo trial at the same time point (p < 0.05). ^#^CHO+AA trial significantly different from placebo trial at the same time point (p < 0.05).

**Figure 6:**
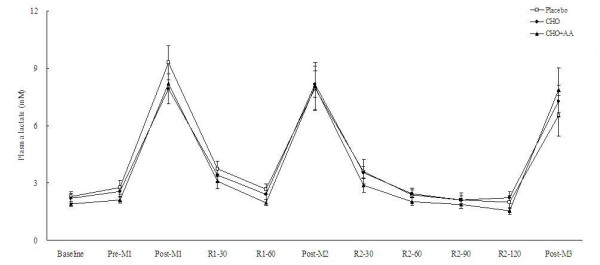
**Plasma lactate concentrations in the 3 trials**. Data were analyzed by using repeated measures ANOVA with time and group as factors. Treatment effect p = 0.546; time effect p < 0.001; interaction effect p = 0.085.

The supplementation of CHO and CHO+AA resulted in significantly lower plasma concentrations of glycerol and NEFA at 90 and 120 min after match 2, as well as immediately after match 3 (Figures 4 and 5). Plasma lactate concentrations were not significantly different among the 3 trials at any time point (Figure 6).

**Figure 7:**
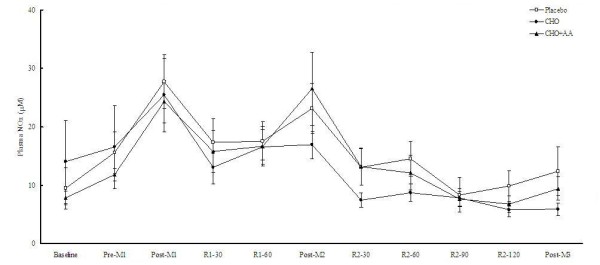
**Plasma NOx concentrations in the 3 trials**. Data were analyzed by using repeated measures ANOVA with time and group as factors. Treatment effect p = 0.533; time effect p = 0.002; interaction effect p < 0.001.

Plasma NO_x _concentrations in the 3 trials were shown in Figure 7. Despite the supplementation of arginine in the CHO+AA trial, there was no significant difference in NO_x _concentration among the 3 trials at any time point.

## Discussion

To our knowledge, this is the first study that investigated the effect of supplementation during a short-term recovery period on the subsequent simulated match performance in combat sports. The results of this study suggested that the supplementation of carbohydrate, with or without additional BCAA and arginine, during the recovery period after two matches had no effect on the performance in the subsequent match in well-trained male college wrestlers.

The few available studies investigating the effect of carbohydrate and protein consumption during the post-exercise recovery period on the performance in the subsequent exercise have provided positive [7,28] and negative [29,30] results. The consumption of carbohydrate and protein during the 4-hr recovery period after glycogen-depleting exercise increased the time to exhaustion in the subsequently exercise at 70-85% $\dot{\text{V}}$ O_2max_, compared to a smaller or same amount of carbohydrate alone [7,28]. The increase in performance may be attributed to higher glycogen resynthesis during the recovery period [7]. However, the carbohydrate-protein supplementation did not show any additional effect compared to isocaloric carbohydrate [28]. On the other hand, consumption of 0.6 g/kg/hr carbohydrate during the 2-hr recovery after a glycogen-depleting exercise resulted in similar time to exhaustion in the subsequent endurance exercise, compared to 1.0 g/kg/hr carbohydrate or 0.6 g/kg/h carbohydrate plus 0.4 g/kg/hr protein [29]. The authors concluded that the additional energy, either in carbohydrate or protein, did not provide additional effect above 0.6 g/kg/hr carbohydrate during the 2-h recovery period [29]. With carbohydrate intake of 0.8 or 1.2 g/kg/hr during the 4-hr post-exercise recovery period, the additional protein showed no effect on the running time to exhaustion at 85% VO_2max _in the subsequent exercise, despite higher insulinemic response [30]. One of the reasons that protein offered no additional benefit may be the higher carbohydrate oxidation rate and similar glycogen utilization rate during the subsequent endurance exercise [31,32]. The aforementioned studies all focused on endurance exercise. For the first time, this study suggested that consumption of carbohydrate or carbohydrate plus BCAA and arginine during the recovery period had no effect on the performance in the subsequent intermittent high-intensity exercise in well-trained wrestlers.

It is generally believed that muscle glycogen resynthesis during the first 4 hours of recovery is proportional to the amount of carbohydrate ingested during the period [33]. While some authors have reported increased rates of muscle glycogen resynthesis following the addition of protein to carbohydrate during recovery periods after glycogen-depleting exercise [17,34], others have found no such effect despite higher insulinemic response induced by protein [35-37]. A recent review suggested that when carbohydrate intake is less than 1 g/kg/hr over the 2-6 hr post-exercise period, the additional protein would increase muscle glycogen resynthesis. On the other hand, when carbohydrate intake is sufficient, i.e. larger than 1 g/kg/hr, the co-ingested protein would not provide additional effect on glycogen resynthesis [38]. Our subjects consumed 0.5 (CHO+AA trial) and 0.6 (CHO trial) g/kg/hr carbohydrate during the recovery period, which may allow the additional protein to result in higher glycogen resynthesis. However, we still found that plasma insulin and glucose concentrations were similar between the 2 trials, indicating that glycogen resynthesis is likely also similar. In agreement to our results, it was reported that consumption of 0.6-0.8 g/kg/hr carbohydrate and 0.25-0.30 g/kg/hr protein resulted in similar glycogen resynthesis rate during a 4-hr post-exercise period compared to the supplementations matched for energy [39] or carbohydrate [40].

The literature on the effects of BCAA on glucose uptake and glycogen synthesis in skeletal muscles has been equivocal [5,41-43]. It has been reported that supplementation of leucine in combination with carbohydrate after exercise resulted in higher post-exercise insulin concentration and greater muscle glycogen recovery in athletes, compared to the same amount of carbohydrate [5]. In addition, oral supplementation of BCAA has been reported to increase glycogen synthase activity in rat skeletal muscles [42]. Leucine has also been shown to increase insulin-independent glucose uptake in isolated rat skeletal muscles through phosphatidylinositol 3-kinase (PI3K) pathway [44]. On the other hand, leucine infusion decreased glucose uptake in human forearm muscles in a dose-dependent manner despite the elevated plasma insulin levels [45]. Infusion of amino acid mixtures containing BCAA and arginine also impaired insulin-stimulated glucose disposal and glycogen synthesis in human skeletal muscles by increasing the inhibitory insulin receptor substrate-1 phosphorylation and decreasing PI3K activity [43,46].

The results on the effect of arginine on post-exercise insulinemic response and glycogen recovery were also mixed. It has been shown that carbohydrate oxidation after exercise was lower after arginine supplementation, indicating the increase of glucose availability for muscle glycogen storage during recovery in well-trained cyclists. However, muscle glycogen resynthesis rate only showed an insignificant trend of increase [47]. Although arginine supplementation after endurance exercise could increase glucose and insulin concentrations during the recovery period in trained athletes [18], it had no additional effect on plasma glucose and insulin concentrations when co-ingested with glucose [48]. Other studies in human subjects have also failed to show the effect of arginine supplementation combined with carbohydrate on post-exercise glycogen recovery, compared to carbohydrate alone [39,48].

The CHO and CHO+AA trial showed significantly lower plasma concentrations of glycerol and NEFA than the placebo trial during the recovery period after match 2. The higher insulin response in the CHO and CHO+AA trials may suppress lipolysis and fat oxidation [49]. The higher plasma NEFA concentration at the onset of match 3 in the placebo trial would lead the subjects to use more fat as the energy source during the match. Indeed, plasma lactate concentration at the end of match 3 tended to be lower in the placebo trial.

All three trials in our study showed higher exercise-induced NO production as NO_x _concentrations were significantly elevated after each match. However, arginine supplementation had no effect on exercise-induced NO production in these well-trained subjects. This result was in agreement with our previous study using similar exercise protocol in college judo athletes [50]. Regular exercise training has been shown to increase basal NO production [51] by stimulating endothelial NO synthase expression and phosphorylation [52]. Therefore, it is possible that these athletes already had higher basal concentration of NO than general population and certain patients [53]. Thus, arginine supplementation did not provide any additional effect on NO production in our subjects.

The lack of effect of carbohydrate supplementation, with or without BCAA and arginine, on the performance of high-intensity intermittent exercise is in contrast to previous studies in which low muscle glycogen content contributed to the development of fatigue in such type of exercise [2,4,54,55]. Although muscle biopsy was not performed, the exercise protocol used in our study would significantly reduce the glycogen content in the working muscles. It has been shown that a single bout of 30-s all-out cycling reduced muscle glycogen by approximately 24% [56]. In addition, muscle glycogen levels were decreased by 19.6-36.4% after 10 to 15 bouts of 6-s all-out cycling, interspersed with 30-s rests [2,57]. Therefore, the decrease in muscle glycogen after our simulated matches would be similar, or even larger, than that in real wrestling matches [22]. Even though the glycogen content in the working muscles would be significantly decreased after two simulated matches in our study, the performance in match 3 was not significantly different from the previous two matches in all 3 trials. One possible explanation is that these experienced wrestlers have the ability to recover quickly from the previous matches. In agreement, it has been reported that grip strength, isometric upper body pull strength, hip and back strength, vertical jump, and isokinetic knee extension peak torque were all generally maintained throughout a 2-day, 5-match freestyle wrestling tournament [23]. A recent study on a 1-day 5-match Greco-Roman wrestling tournament also revealed that these parameters were generally maintained through the first three matches [24]. The length and work:rest ratio of the simulated match in this study resemble real wrestling competitions. It also resulted in the similar post-match plasma lactate concentrations to those in the literature [22,58]. Therefore, it is possible that these well-trained wrestlers are adapted to this type of exercise and able to recover within 1 to 2 hours of rest. Furthermore, well-trained endurance athletes can also maintain the time to fatigue in intermittent exhaustive cycling exercise despite lower muscle glycogen levels [59]. Therefore, the well-trained wrestlers in this study may be able to maintain the performance in the three matches with or without the supplementation.

Another unique characteristic of this study is that subjects consumed a carbohydrate-rich breakfast before the exercise began. In previous studies investigated the effect of ingestion of carbohydrate and protein (or amino acids) during post-exercise recovery, subjects were mostly at an overnight fasted state. It appears that the carbohydrate in the breakfast was sufficient to maintain the euglycemic states throughout the entire study period, even in the placebo trial. Although the breakfast might mask the potential benefit of the supplementation during the recovery period, it more closely reflects the real-life behavior of athletes as they rarely participate in matches in a fasted state.

The amount of BCAA consumed in this study, 7 g in a 70-kg subject, was similar to the 6.5-15.8 g dosages ingested before exercise in the literature [60-62]. The amount of arginine consumed in this study, 7 g in a 70-kg subject, has been shown to result in a significant improvement of flow-mediated vasodilatation [63]. In addition, it has been suggested that post-exercise supplementation of 0.3-0.5 g total protein/kg/hr could produce higher insulinemic responses [38]. Since whey protein hydrolyate containes approximately 13.4% amino acids as BCAA and arginine [17], we selected 0.1 g amino acids/kg/hr in this study.

A limitation of this study is that muscle biopsy was not performed because it would interfere with the performance in the subsequent exercise. Future studies with modified protocols may allow the biopsy procedure and further clarify the effect of BCAA and arginine on post-exercise glycogen recovery. Another limitation of this study is that inflammatory response was not measured. Strenuous exercise such as the simulated match in this study could result in significant inflammatory response and muscle damage. However, there was no significant difference in plasma concentrations of creatine kinase and lactate dehydrogenase at the baseline among the 3 trials (data not shown). It is reasonable to assume that the 2-week period between each trial is sufficient for the subjects to recover completely. The other mechanisms that may affect the performance in multiple wrestling matches, such as neuromuscular and/or psychological fatigue, were not investigated in this study and could be involved in future studies.

## Conclusions

In conclusion, this study suggested that supplementation of carbohydrate with or without BCAA and arginine during the post-match period did not provide additional effect on the performance in the following simulated match in well-trained male wrestlers when a carbohydrate-rich breakfast was eaten. It is possible that factors other than muscle glycogen content contribute to the performance in multiple bouts of high-intensity intermittent exercise. It is also possible that experienced wrestlers have the ability to recovery quickly from previous matches with or without supplementation. Furthermore, BCAA and arginine did not provide additional insulinemic effect when given after high-intensity intermittent exercise.

## List of abbreviations used

AUC: area under curve; BCAA: branched-chain amino acids; GLUT: glucose transporter; NEFA: non-esterified fatty acid; NO: nitric oxide; PI3K: phosphatidylinositol 3-kinase.

## Competing interests

The authors declare that they have no competing interests.

## Authors' contributions

TRJ and CLW designed the study and assisted the manuscript preparation. CMC and WH were responsible for conducting the study, including subject recruitment, biochemical measurements, and data analysis. SHF assisted the design of the study and manuscript preparation. CKC was responsible for statistical analysis and manuscript preparation. All authors read and approved the final manuscript.
